# Five-years surveillance of invasive aspergillosis in a university hospital

**DOI:** 10.1186/1471-2334-11-163

**Published:** 2011-06-08

**Authors:** Karolin Graf, Somayeh Mohammad Khani, Ella Ott, Frauke Mattner, Petra Gastmeier, Dorith Sohr, Stefan Ziesing, Iris F Chaberny

**Affiliations:** 1 Institute for Medical Microbiology and Hospital Epidemiology, Hannover Medical School, Hannover, Germany; 2 Institute for Hygiene, University Hospital Witten-Herdecke, Campus Köln-Merheim, Germany; 3 Institute of Hygiene and Environmental Medicine, Charité - University Medicine Berlin, Germany

**Keywords:** surveillance, invasive Aspergillosis, epidemiology, pathology

## Abstract

**Background:**

As the most common invasive fungal infection, invasive aspergillosis (IA) remains a serious complication in immunocompromised patients, leading to increased mortality. Antifungal therapy is expensive and may result in severe adverse effects.

The aim of this study was to determine the incidence of invasive aspergillosis (IA) cases in a tertiary care university hospital using a standardized surveillance method.

**Methods:**

All inpatients at our facility were screened for presence of the following parameters: positive microbiological culture, pathologist's diagnosis and antifungal treatment as reported by the hospital pharmacy. Patients fulfilling one or more of these indicators were further reviewed and, if appropriate, classified according to international consensus criteria (EORTC).

**Results:**

704 patients were positive for at least one of the indicators mentioned above. Applying the EORTC criteria, 214 IA cases were detected, of which 56 were proven, 25 probable and 133 possible. 44 of the 81 (54%) proven and probable cases were considered health-care associated. 37 of the proven/probable IA cases had received solid organ transplantation, an additional 8 had undergone stem cell transplantation, and 10 patients were suffering from some type of malignancy. All the other patients in this group were also suffering from severe organic diseases, required long treatment and experienced several clinical complications. 7 of the 56 proven cases would have been missed without autopsy. After the antimycotic prophylaxis regimen was altered, we noticed a significant decrease (p = 0.0004) of IA during the investigation period (2003-2007).

**Conclusion:**

Solid organ and stem cell transplantation remain important risk factors for IA, but several other types of immunosuppression should also be kept in mind. Clinical diagnosis of IA may be difficult (in this study 13% of all proven cases were diagnosed by autopsy only). Thus, we confirm the importance of IA surveillance in all high-risk patients.

## Background

As the most common invasive fungal infection, invasive aspergillosis (IA) remains a serious complication in immunocompromised patients [1-3]. The number of patients at risk is increasing due to new intensive chemotherapy regimens and a growing number of stem cell and solid organ recipients. Because *Aspergillus spp*. are widely found in the environment, water, soil and in decomposing plants, spores can be easily inhaled and may then cause invasive pulmonary disease. The IA incidence in organ transplant recipients may be as high as 5%; mortality rates of up to 80% have been reported [4]. Antifungal therapy is expensive and adverse effects can be severe [5]. Thus, the Centers for Disease Control and Prevention (CDC) recommend the need for surveillance of pulmonary IA in hospitals [6].

Unfortunately, surveillance of IA is difficult to perform and various methods exist aiming to detect cases of IA using computerized data or chart review. Usually specially trained staff (qualified data review committee) are required to correctly evaluate cases [7-9].

The aim of the five year surveillance study presented here was to obtain an overview of IA cases in the entire university hospital using a standardized surveillance method.

## Methods

### Facility

The study was conducted at Hannover Medical School, a 1,419-bed university hospital with 50,000 inpatient admissions per year. A large proportion of the patients are severely immunocompromised because Hannover Medical School focuses on transplantation of solid organs, stem cells and bone marrow. From 2003 through 2007, about 2,180 organ transplantations and 625 stem cell transplantations were performed. Intensive care units (ICUs) and hematologic transplant units are equipped with HEPA air filtration and have positive air pressure (reverse isolation) rooms.

### Construction works

Hannover Medical School was undergoing major construction and renovations during the entire study period. Work was also being carried out in patient care areas in clinical buildings. ICUs and non-ICUs were affected in equal measure. Units were only closed completely for extensive construction work. In order to prevent spread of *Aspergillus *spores, we introduced the following prevention measures as recommended by the CDC: (a) work sites were isolated with impermeable barriers, (b) specific routes were defined for transportation of materials, machines and building site workers, and (c) cleaning of units was intensified.

### Air sampling

At Hannover Medical School, air sampling is performed routinely twice a year on ICU and hematologic-oncologic wards and was also performed throughout the duration of construction works.

Air sampling was undertaken with one of the most frequently used air samplers, the Reuter centrifugal sampler (RCS High flow Air Sampler 35318, Biotest Diagnostics Corporation, Denville, NJ, USA).

### Surveillance method and case definition

The Department of Hospital Epidemiology and Infection Control reviewed the following data and classified IA cases as "proven", "probable", or "possible" according to European Organisation for Research and Treatment of Cancer (EORTC) criteria [10,11].

The first and most important step in the surveillance method employed here involved a microbiology database query using the keyword *Aspergillus *to retrieve possible cases of IA in microbiological samples.

Data of all patients were reviewed for the following indicators:

(a) Microbiology: An electronic alert was sent by the microbiological laboratory as soon as there were at least two positive results: either a positive culture for *Aspergillus *spp. (detection of septated hyphae) or serum found positive for *Aspergillus *antigen using the galactomannan Platelia *Aspergillus *test with a cut-off of 0.5 (Bio-Rad). The positive results were documented in a table via electronic retrieval, and then evaluated by our infection control team.

(b) Pathology: Proof of mould infection in histological samples or in autopsy. Electronic retrieval involved searching for the keywords: mould, hyphae, mycosis, invasive mycosis, invasive aspergillosis, aspergillus. Medical reports including these keywords were evaluated by our infection control team. (c) Antimycotics: Use of at least one of the following substances was documented: amphotericin B, caspofungin, voriconazole, and flucytosine. The antimycotics are documented in an electronic program operated by the in-house pharmacy. The charts of all the patients who had received at least one of the antymycotics were evaluated by our infection control team. The reason for application (prophylaxis vs. therapy) as well as the type of application (oral vs. intravenous) was also recorded.

In addition: Radiological findings that raised suspicion of mould infection (halo sign, air-crescent sign or other signs suggestive of invasive sinusitis). Patients showing one of the three symptoms were screened for radiological signs using the keywords mould infection, aspergillosis, invasive aspergillosis, fungal infection, halo sign, crescent sign. Medical reports including these keywords were later evaluated by our infection control team.

Most of these parameters were collected from the hospital's patient data documentation system. Only autopsy reports had to be screened separately as they are not yet available electronically in our system

We excluded cystic fibrosis patients who had not yet undergone lung transplantation because they are often colonized without invasive infection. Patients with invasive candidiasis were also excluded. All other inpatients were included in the study. A case of IA was classified as being likely to have been nosocomially acquired if clinical symptoms appeared more than seven days after admission with at least one negative respiratory culture before the positive index sample [7]. Cases with an infection occurring within seven days of admission were defined as possibly nosocomial.

Cases positive for one or more of these indicators were then further evaluated using EORTC criteria including detailed chart review and audits with clinical personnel [10,11]. We collected demographic data (age, gender), potential host factors that might predispose for IA, and clinical, microbiological and histological diagnoses.

Infection was defined as early if occurring within the first 100 days of transplantation, and as late if detected later than 100 days.

As the Department of Hospital Epidemiology and Infection Control, we are entrusted with analysis of data for surveillance purposes, and obtained full permission from our hospital's administration to use of all the data generated.

### Statistical analysis

Monthly or annually incidence densities of nosocomial IA/1000 patient days were calculated as the number of patients with any IA event in a given month or year, divided by the number of patient days for that month or that year and multiplied by 1000.

Mortality refers to in-hospital mortality only, because there was no post-discharge surveillance of patients and no surveillance of outpatients.

Time trends of the five year surveillance programme were investigated. Differences of annual incidence densities were calculated using exponential MLE-Test. p values < 0.05 were considered statistically significant. To show use of antifungal therapy, we collected all data on antifungal therapy, calculated the daily defined dosages (DDD) per year and correlated them with cases.

## Results

### Characteristics of all IA cases

During the study period, there were 234,095 admissions to Hannover Medical School.

At least one of the pharmacological, pathological or microbiological indicators (in addition clinical and radiological signs) was reported in 704 patients. Only these cases had do be reviewed in more detail. Of this number, 214 cases of IA were confirmed as either proven (56; 26%), probable (25; 12%) or possible (133; 62%). 35 proven cases and 3 probable cases (overall 44% of all proven and probable cases) were classified as health-care associated. The number of indicators and the number of cases resulting are shown in table 1.

**Table 1:** Cases found through indicators and IA cases resulting (with each method)

	all cases	cases with one indicator		cases with two indicators			cases with three indicators
		M*	PA*	M, PH*	M, PA*	PH, PA*	M, PH, PA*
**no IA**	490	231	6	253	-	-	-

**proven IA**	56	3	6	17	13	2	15

**probable IA**	25	6	-	17	-	1	1

**possible IA**	133	45	-	88	-	-	-

* M = Microbiological indicator, PA = Pathological indicator, PH = Pharmacological indicator

58 of the 214 IA cases died (crude mortality rate 27%). Mortality rates differed depending on the classification of cases: 25 of 56 proven cases (46%), 11 of 25 probable cases (44%), and 22 of 133 possible cases (17%).

The incidence of nosocomial IA (proven and probable cases) was 1.85% in organ transplanted patients (mostly from cardiothoracic surgery ICUs) and 0.97% in stem cell transplanted patients. We detected no *Aspergillus *outbreaks, nor did we find any correlation between the onset of IA and seasonal variation or construction works. Incidence densities declined when comparing data on invasive *Aspergillosis *from 2007 with those of 2003 (p = 0.0004). Changes in incidence densities between the years are shown in table 2.

**Table 2:** Incidence densities of IA

Year	# of cases (proven and probable)	# of patient days	incidence density (per 100,000 patient days)	95% CI	p-value (vs. 2003)
2003	32	391.445	24	5.59; 11.54	-

2004	16	407.007	15	2.25; 6.38	0.008

2005	15	407.644	6	2.06; 6.07	0.427

2006	7	415.980	5	0.68; 3.47	0.044

2007	11	431.954	4	1.27; 4.56	0.804

### Hospital-acquired cases of IA

**Figure 1:**
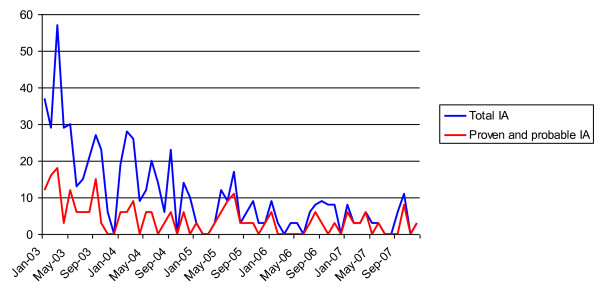
**Monthly incidence of invasive Aspergillosis (IA)**.

**Figure 2:**
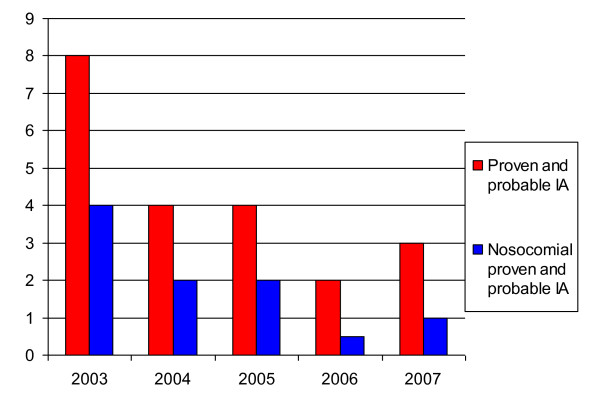
**Yearly incidence densities of invasive Aspergillosis (IA)**.

The air outlets of indoor air supply systems were checked regularly for presence of mould spores. Furthermore, the concentration of particles, bacteria and fungi was routinely measured inside and outside the building with no increase being noted during this study. Incidence rates of IA cases are shown in Figure 1 and 2.

### Characteristics of the 81 proven and probable IA cases

The median age of proven and probable IA cases was 51 years (50 years on average, 25^th ^percentile: 34 years; 75^th ^percentile: 63 years). There were 59 male and 32 female cases (male/female ratio 2.5). Underlying diseases of the proven and probable IA cases were (a) hematological diseases (n = 26; 21%), (b) severe chronic diseases of the lung (n = 30; 24%), (c) liver diseases (n = 20; 16%), (d) solid tumors (n = 10; 12%), and (e) chronic renal diseases (n = 7; 6%).

Detailed data on underlying diseases and distribution of organ transplantations for IA cases are shown in table 3 and 4.

**Table 3:** Underlying diseases for all confirmed cases of invasive aspergillosis

underlying diseases	proven + probable n = 81	possible n = 133	total (%) n = 214
hematological diseases	17 (38%)	55 (41%)	72 (34%)

organ transplantation	39 (49%)	55 (41%)	94 (44%)

single lung transplantation	1	2	3
double lung transplantation	13	23	36
liver transplantation	9	12	21
heart transplantation	4	2	6
heart-lung transplantation	3	3	6
renal transplantation	4	0	4
stem cell transplantation	4	13	17
pancreas transplantation	1	0	1

liver diseases	12	14	26

lung diseases	15	31	4

kidney diseases	4	4	8

heart diseases	4	1	5

heart and lung diseases	3	5	10

pancreas diseases	1	0	1

malignancy (solid tumor)	10 (22%)	6 (5%)	15 (7%)

human immunodeficiency virus	0	0	0

immunosuppressive treatment	73 (85%)	117 (88%)	190 (89%)

**Table 4:** Underlying diseases for proven and probable cases of invasive aspergillosis (IA) without transplantation or primary immunosuppression

underlying diseases/therapy	proven + probable IA n = 81	total (all proven and probable cases) (%)
tumor diseases	10	12.3%

colorectal cancer (colectomy, rectal resection)	4	4.9%
gastric cancer (gastrectomy)	2	2.5%
cholangiocarcinoma (tumor resection)	1	1.2%
hepatocellular carcinoma	2	2.5%
renal carcinoma (nephrectomy)	1	1.2%

other diseases	26	32.5%

gastroenterologic diseases	10	12.3%
necrotizing enterocolitis	1	1.2%
necrotizing pancreatitis	2	2.5%
chronical hepatitis B infection	3	3.7%
liver cirrhosis	3	3.7%
citrullinaemia	1	1.2%

prematurity	1	1.2%

heart diseases	4	4.9%
congenital heart disease	1	1.2%
dilatative cardiomyopathy	2	2.5%
endocarditis	1	1.2%

vascular diseases	1	1.2%
type B dissection (stent implantation)	1	1.2%

renal diseases	4	6.2%
renal insufficiency	4	4.9%

other diseases	5	6.2%
multiple trauma	3	3.7%
sinusitis maxillaris	2	2.5%

39 patients (48%) had transplantations (35 organ transplantations and 4 stem cell transplantations).

44 of the proven and probable cases (44/81) were categorized as being of likely nosocomial origin, 19 cases (19/81) were possible nosocomial cases.

28 of the likely nosocomial cases (28/39) were defined as early infection (up to 100 days after transplantation); 11 likely nosocomial cases (11/39) showed late infection (detected > 100 days).

We identified 10 patients suffering from some kind of malignant tumor and a further 26 patients with chronic organ diseases. Detailed information on the underlying diseases of these patients is given in table 4. All patients suffering from chronic organic diseases had received long term treatment, or had experienced severe complications (e.g. insufficiency of anastomoses, re-operations, or peritonitis). However, they had neither undergone transplantation, nor were they immunosuppressed..

The mortality rate for all proven and probable cases was 45%. Seven of the 11 proven IA cases who had not received prior antifungal therapy died (64%). 72 proven/probable cases (89%) had been treated with immunosuppressive therapy prior to first signs of IA (e.g. high dose steroidal therapy, cyclosporine, or chemotherapy). Most of the cases were detected in review of microbiology and pharmacy reports (58 cases, 72%). 34 cases (42%) had positive pathology results.

### Microbiological data

Of the 81 proven and probable cases, *Aspergillus sp*. was identified in a clinical sample of 55 cases (68%). 34 cases (57%) had positive pathological results. 16 proven IA cases (29%) were detected by autopsy, seven of which did not have any other advance symptoms, meaning that 13% of all proven IA cases would have been missed without subsequent autopsy. Among the 133 possible cases, 49 had positive clinical samples (table 5).

**Table 5:** Classification of invasive aspergillosis by positive microbiological culture

positive microbiological samples/ alerts	proven n = 56	probable n = 25	possible n = 133	total n = 214 (%)
microbiological culture	41	17	49	107 (50%)

broncho alveolar lavage	20	14	27	61 (29%)

bronchial secretion	13	7	26	46 (21%)

tracheal secretion	14	9	26	40 (19%)

### Data on antifungal therapy

**Figure 3:**
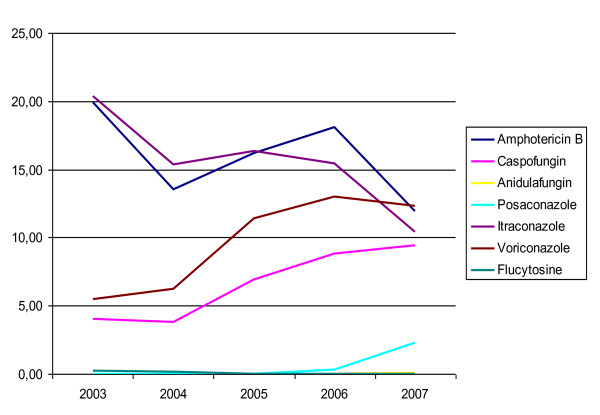
**Usage of antifungal therapy calculated per 1000 Patient days**.

As shown in Figure 3, the antifungal therapy regimen changed during the investigation period. Overall use of antimycotics increased (from 32,705 DDD in 2003 to 36,327 DDD in 2007). However, use of flucytosine was terminated in 2005. Instead, posaconazole was introduced in 2006, with consumption increasing by factor 7 in 2007 (from 131 to 998 DDD). Anidulafungin was introduced in 2007 (27 DDD). A reduction in use of amphotericin B (from 7786 to 5164 DDD) and itraconazole (from 7,964 to 4,489 DDD) became apparent during the study, while use of caspofungin (from 1,582 to 4,080 DDD) and voriconazole (from 2,141 to 5,311 DDD) increased.

During the investigation period, the departments of hemato-oncology and cardio-thoracic surgery implemented standardized long-term prophylaxis for all transplanted patients (Initially 1 × 200 mg, followed by 2 × 200 mg itraconazole daily). Hence, pharmacological reports did not add to detection of further cases of IA. Only cases found in pharmacological alerts were evaluated by our infection control team. Not a single case of proven, probable or possible IA could be confirmed. All the cases detected were positive for at least one of the other alerts.

## Discussion

Surveillance of IA is important because it is the only way minor trends and major changes in incidence density can be detected over time. Furthermore, it may help identify IA cases in patients with various coexisting organ diseases (which can make diagnosis difficult). Thus, the CDC and several other experts in the field of IA prevention recommend surveillance. Unfortunately, however, so far, there is no standardized epidemiological method available to do so.

In this investigation, we used a simplified surveillance alert system that relied on three indicators to detect possible cases of IA. This process allowed to screen for IA in the entire hospital (not only in hematology) and to detect possible clusters of cases especially in other disciplines. Our data show that IA may occur in all types of medical departments, and in all kinds of patients with severe underlying diseases.

Our surveillance system was implemented in 2003 and ended in 2007, which is why we did not apply the revised EORTC criteria published later in 2008. Application of the revised criteria in future studies covering a different set of patients (e.g. patients with solid organ transplantations, hereditary immunodeficiencies and connective diseases) should by all means be considered, bearing in mind, however, that the category of proven invasive fungal disease can now apply to any patient, regardless of whether the patient is immunocompromised, whereas probable and possible categories are proposed for immunocompromised patients only. Because we would wish to include all patients the revised EORTC criteria might [12] not be helpful. Definition of nosocomial IA remains difficult. A better understanding of early events related to IA onset will help improve treatment of this disease, for which the prognosis remains negative. Thus, in the study presented here, infections were defined as early if occurring within 100 days of transplantation and as late if detected later than 100 days. To our knowledge, a clear definition of nosocomial infection can only be given if there is at least one negative respiratory culture after admission [7]. Cases with an infection occurring within seven days after admission without a negative culture prior to a positive result still remain uncertain and were thus defined as possibly nosocomial

The majority of patients with IA had a history of transplantation (visceral, cardiac, and thoracic surgery) (94; 44%). Only 72 (34%) of the IA cases were cared for in hematology or in infectious diseases units. This unexpected distribution has been reported previously [13]. Our findings therefore implicate that IA surveillance needs to be extended and should comprise the entire hospital including all high risk units [9]. Accordingly, alert systems that might already be in place for hematological patients should be expanded to patients after organ transplantation and patients with other severe chronic diseases.

Compared with the literature, we noticed a significant decrease in the incidence density of IA and low rates from 2005 through 2007 [13], which probably reflects changes in antimycotic prophylaxis policy. During 2003 and 2004, antimycotic prophylaxis was implemented in several high risk units. For example, the departments of hemato-oncology and the cardio-thoracic surgery implemented a standardized prophylaxis protocol for transplanted patients, which led to a significant decrease in the number of cases.

Pharmacological reports did not lead to detection of additional cases of IA. Not a single case of proven, probable or possible IA could be confirmed by means of this indicator alone. All the cases detected were positive for at least one of the other indicators. However, we suggested maintaining the indicator for surveillance purposes because it was an important point of discussion between clinicians and infection control team. During prospective surveillance, this indicator was helpful in encouraging clinicians intensify microbiological diagnostics, which was essential for categorization of IA.

In terms of case investigation, it is important to emphasize that chart review and audits with clinical personnel is crucial for all potential cases presenting with symptoms matching one of the indicators we were using. Other authors who also use an active surveillance system report that although computer-assisted surveillance may be quite sensitive, it is not very specific [14]. This is why we established the method of surveillance presented here. However, extensive chart review is only necessary if patients present a positive indicator. This way, we had the possibility to quickly screen 234,095 patients admitted during 2003 through 2007, but only 704 of that number required a more detailed review. There are other simplified surveillance methods in place, but they showed to be suitable for special patient groups only, while further clarification often remains impossible due to the lack of additional clinical data [14].

Reliance on microbiological data alone to identify cases may lead to ascertainment bias, because many cases of IA are diagnosed radiographically, by pathologic investigation or by clinical suspicion. Our method, which includes three indicators that are as far as possible standardized, may also be suitable for use in other institutions, and might even facilitate inter-institutional comparison of incidence rates in special patient groups or in all patients.

The EORTC criteria used in our study represent the first established international criteria for definition of IA. They were primarily developed for immunocompromised patients with cancer and hematologic stem cell transplantation; however, as shown here, they are also applicable to other patient groups. Nevertheless, caution should be exercised in applying the criteria. Only 56 (26%) of the 214 cases of IA investigated were proven, 25 (12%) were probable, whereas, according to these criteria, the majority (133; 62%) were possible cases. With regard to the possible cases, the true diagnosis remains to some extent uncertain. Clinical diagnosis, microbiological findings and the pathologic results may differ. If certain data - especially microbiological and pathologic results - are missing, the correct diagnosis can also easily be missed. It is an important fact that the number of respiratory samples taken may also influence the number of cases. We noticed this effect in our surveillance study because we included the whole hospital over a long time, and different culturing regimes (e.g. routine culturing of tracheal secretions or screening at admission in some ICUs) were used in various departments at our facility.

For inter-institutional comparison of incidences it is important to calculate the rates of possible and probable nosocomial cases. In this study, 32 of the proven cases (57%) had positive pathology results, 16 (29%) were detected in autopsy and seven cases did not match any other alerts, meaning that 13% of all proven cases would not have been detected without autopsy. From 2003 through 2007, autopsy rates at our hospital ranged between 14.1% and 20.9%, and decreased during the investigation period. Thus, we surmise that some cases were missed because of lacking autopsy. In Germany, autopsy rates have decreased steadily since 1980 and are the lowest in the world today [15]. Thus, studies carried out under these circumstances may underestimate the actual problem.

We documented antifungal therapy such as amphotericin B, voriconazole and caspofungin. In our study, reports from the in-house pharmacy did not help detect diseased patients. However, they can help identify risk patients. On the one hand, many patients received antifungal treatment although we found that the criteria were uncertain for IA. On the other hand, antifungal drugs are used for prophylaxis, especially in transplanted patients [4]. Obviously clinicians are often uncertain about the indication for antimycotic therapy or aim to prevent colonization in the first place [16]. IA is often seen in patients with different severe chronic organ diseases, making it difficult to get the diagnosis right without hard microbiological and/or pathological data. Even under adequate antifungal therapy, mortality rates of between 30% and 80% are reported for IA, and antifungal drugs might be used for prophylaxis more often than for therapy [17]. It is worth noting that pharmacological data may be supportive for detection of IA cases, although they are of little help for direct detection. During prospective surveillance these data may serve as a first step for contacting clinicians, and for discussing diagnostic procedures, infection control measures and treatment options. Patients undergoing immunosuppressive therapy have often been reported to be "high risk" patients for acquiring IA, and especially patients suffering from hematological diseases and/or with stem cell transplantation [18,19]. We are able to confirm these findings since we also found a large number of IA in transplanted patients, but also in patients with solid organ malignancy tumors. Two patients were undergoing chemotherapy and three other patients were receiving high dose steroidal therapy. Due to the rather small number of patients we cannot define the presence of a solid tumor as an independent risk factor; however, we do recommend that patients with malignancies should be included in a surveillance programme. In our study, the incidence of likely nosocomial IA (proven and probable cases) was 1.85% in organ transplanted patients and 0.97% in stem cell transplanted patients. In other studies, the incidence of IA was evaluated as a frequency of 1 to 28% for patients with allogenous bone marrow transplant graft [14,19]. IA incidence rates for solid organ transplant patients varied depending on the type of transplanted organ: 2 to 18% for lung [20-22], 1.5 to 10% for liver [23,24] and 1.3 to 7% for heart [25-27].

The concentration of Aspergillus spores in the surrounding air may also influence the likelihood of subsequent infection. Fortunately, during the 60 months of epidemiological surveillance, no outbreaks or clusters and no association between the onset of IA and seasons was observed.

In our study, crude mortality (45%) for proven and probable IA cases was relatively low compared with the findings of others (50-100%) [2,26,27]. Since patients with IA often suffer from severe underlying diseases, we cannot say with full certainty whether IA was causative for or at least contributed to their fatal outcome. Thus, the role of IA sometimes remains unknown. Some patients may be colonized with *Aspergillus *spp. for a long time before developing terminal IA. In this case, IA may rather serve as a sign of the severity of immunosuppression due to some other illness. In our study (34 deceased patients), the time frame from IA diagnosis to death was only eight days in median, however, culturing of the micro-organism may take several days. Thus, clinicians may not have enough time to choose and introduce the appropriate treatment.

Our data show that surveillance of IA should be implemented in all areas of the hospital that care for severely ill patients. A multidisciplinary working group of infection control staff, microbiologists, pharmacologists and clinicians would give a straightforward approach to IA surveillance and help clinicians detect all kinds of high risk patients and cases of IA more easily and at an earlier stage. Our five year survey shows a high frequency of IA cases in patients hospitalized in units other than hematology. Thus, our study emphasizes the importance of IA surveillance, not just in hematology units; rather, surveillance should be expanded to cover all high-risk units (ICUs, respiratory care, infectious diseases and internal medicine units, in particularly).

## Conclusions

This study provides an overview on the incidence of IA cases in a university hospital through use of a standardized surveillance system. It confirms previous findings on patient groups, prophylaxis and treatment options and provides new insights into certain aspects of IA surveillance covering the entire hospital.

Only the charts of 704 of 234,095 patients admitted (0.003%) over a five year time frame (2003 to 2007) required more intensive reviewing as those patients had at least one special indicator (microbiology, pathology, pharmacology). Yet, diagnosis remains difficult because 13% of the cases were diagnosed by autopsy only.

One should keep in mind that so-called "high risk" patients for IA are not only those undergoing transplantation, but all other patients receiving immunosuppressive therapy. Thus, this type of surveillance approach should be performed hospital-wide and should include all patients that are potentially at increased risk of IA.

## Abbreviations

CDC: Centers for Disease Control and Prevention; DDD: daily defined dosages; EORTC: European Organisation for Research and Treatment of Cancer; HEPA: high efficacy particulate air; IA: invasive aspergillosis; ICUs: intensive care units

## Competing interests

The authors declare that they have no competing interests.

## Autor's Contributions

KG: data collection and evaluation, manuscript draft. SM: data collection and evaluation. EO: performance of statistical analysis and evaluation. FM: data collection and evaluation in transplanted patients, supported manuscript draft. PG: participation in the design of the study and supervision, supported manuscript draft. DS: performance of statistical analysis and evaluation. SZ: generation of microbiological data. IC: study conceive, participation in the design of the study, evaluation and coordination. All authors read and approved the final manuscript.

## Pre-publication history

The pre-publication history for this paper can be accessed here:

http://www.biomedcentral.com/1471-2334/11/163/prepub
